# Study protocol for a cluster randomized controlled trial to evaluate a referral strategy for axial spondyloarthritis in young primary care patients with chronic low back pain; an impact study

**DOI:** 10.1186/s12891-016-1132-6

**Published:** 2016-07-12

**Authors:** Lonneke van Hoeven, Yvonne Vergouwe, Bart W. Koes, Johanna M. W. Hazes, Angelique E. A. M. Weel

**Affiliations:** Erasmus MC, Department of Rheumatology, Wytemaweg 80, 3015 CN Rotterdam, The Netherlands; Maasstad Hospital, Department of Rheumatology, Maasstadweg 21, 3079 DZ Rotterdam, The Netherlands; Erasmus MC, Department of Public Health, Wytemaweg 80, 3015 CN Rotterdam, The Netherlands; Erasmus MC, Department of General Practice, Wytemaweg 80, 3015 CN Rotterdam, The Netherlands

**Keywords:** Axial Spondyloarthritis, Chronic Low Back Pain, Primary Care, Referral, Cost-effectiveness, Cluster Randomized Trial

## Abstract

**Background:**

Axial spondyloarthritis (axSpA) is a disabling inflammatory joint disease with chronic low back pain (CLBP) as leading symptom. Recognizing axSpA in the large amount of CLBP patients is difficult for general practioners (GP). This evaluation aims to assess the effect of a referral strategy for axSpA in young primary care patients with CLBP by comparing the use of the strategy with usual care. The effect is measured at three different levels; by patient reported outcomes (the clinical effect), process and costs evaluation.

**Methods/Design:**

This study design is a cluster randomized controlled trial with GP as clusters. GPs throughout the Netherlands are invited to participate and randomized to either the intervention or the control group. Patients from participating GPs are invited to participate if they have ever been registered with low back pain, without radiation (ICPC L03) and aged 18–45 years. To be included in the study, patients need to have current low back pain and chronic low back pain (>12 weeks). In the intervention arm a referral strategy for axSpA will be applied in CLBP patients, in the control arm care as usual will be provided for CLBP patients. The referral strategy consists of four easy to use variables. All are questions about the back pain complaints of the patients. Data is prospectively collected in an online database at baseline (T0), 4 months (T1), 12 months (T2) and 24 months (T3). After time point T1 (4 months) patients from the control group will also receive the intervention i.e. the application of a referral strategy for axSpA. The effect of the referral strategy is measured at three different levels, by patient outcomes (e.g. pain scores, quality of life), process measures (e.g. number of axSpA diagnoses by rheumatologists) and by costs (work productivity and health care resources use). Our primary outcome is the Roland Morris Disability Questionnaire after 4 months, secondary outcomes are pain and quality of life. Costs will be assessed before and after the use of the referral strategy, to estimate if the use of the strategy will lead to a reduction in health care costs and improvement in work participation.

**Discussion:**

It is anticipated that using the axSpA referral strategy for primary care CLBP patients will increase the quality of life of CLBP patients, will result in more (correct) diagnoses of axSpA by the rheumatologists, and will be cost-effective. Ultimately, the results of this study may contribute to the startup of a national implementation of the axSpA referral strategy to identify timely CLBP patients with axSpA.

**Trial registration:**

NCT01944163, date of registration; September 6, 2013 (Clinicaltrials.gov)

## Background

Low back pain (LBP) is one of the most common musculoskeletal disorders affecting up to 85 % of the adults at some point in their lives [1]. In 10–28 % of the patients the pain persists for more than 12 weeks and becomes chronic [2]. On top of the high prevalence, LBP is the leading cause of years lived with disability (YLD). The YLD of low back pain is higher than the YLD of e.g. major depressive disorders, anemia, chronic obstructive pulmonary disease and diabetes [3].

One of the possible causes of chronic low back pain (CLBP) is axial spondyloarthritis (axSpA) which is a heterogeneous inflammatory joint disease. Two recent studies showed a prevalence of axSpA among young (18–45 years) CLBP patients between 16 % and 24 % [4, 5]. Recently the focus of axSpA is on early diagnosis considering treatment is more effective in patients with short symptom duration [6]. For an early diagnosis of axSpA by rheumatologists, early recognition in primary care is important. However recognition of axSpA is difficult because specific signs or symptoms do not exist [7]. Moreover, in the current CLBP guidelines for GPs no referral guidelines for axSpA are included [8].

Within the field of rheumatology several models to identify patients at high risk for axSpA have been published, these models combine multiple predictors, such as clinical symptoms, patients’ characteristics and test results to estimate the probability of the disease. Almost all published referral models are tested in a pre-selected population with a high prior probability of axSpA. Only one referral strategy is developed and externally validated in a primary care CLBP population, the CaFaSpA referral rule [4, 5]. This low cost referral rule is easy to use and consists of four variables, all variables are questions. The GP can ask these questions while taking a patient’s history.

After development and external validation of a referral rule the next step before application in daily practice is to investigate the impact of the referral rule [9, 10]. Since the CaFaSpA referral rule can identify axSpA patients, it is worthwhile to perform an impact analysis to determine its effect in primary care.

### Objective of the evaluation

This study entails a clinical effect, process and cost evaluation of using the axSpA referral strategy for primary care CLBP patients. The study aims to determine to what extent use of the rule, in comparison with usual care, leads to less disability and pain and improved quality of life in CLBP patients. Second, it evaluates if the referral strategy leads to more diagnosis of axSpA and finally health care costs and work participation will be compared before and after the application of the referral strategy.

## Methods

### Design

The study uses a cluster randomized controlled trial design which is carried out in the primary care setting in the Netherlands. Sixty primary care practices will be randomized to either the intervention or the control (usual care) group. Each cluster contains the GPs from one practice and their included patients.

### General practices

GPs at the surrounding areas of participating Dutch rheumatologists will be invited to participate by an invitation letter. Two weeks after this invitation letter a member our research team will call the GP to assure if the GP was interested in participating. The only exclusion criteria for GPs is not using the International Classification of Primary Care (ICPC) coding system for their patients, as patients will be selected from the GP practice using the ICPC system. The ICPC is the standard for coding and classification of signs, symptoms and complaints in general practice. The Dutch ICPC is managed and maintained by the Netherlands Society of General Medical Practitioners (NHG). At present, most general practice information systems use the ICPC codes.

### Recruitment of patients and eligibility criteria

Patients will be recruited from participating practices by searching their records for patients with ICPC L03 and aged between 18 and 45 years. The recruitment of patients is the same for GPs randomized to the intervention as for GPs randomized to the control group. All selected patients will receive a letter from their GP briefly explaining the study and asking the patient to respond using the attached return form. If the patient does not respond to the invitation within 4 weeks, a second invitation letter will be sent.

The inclusion criteria are:

- Age 18–45 years
- Ever registered with low back pain, without radiation ICPC L03
- Current low back pain
- ≥12 weeks low back pain

The exclusion criteria are:

- A clear explanation for the back pain (like a trauma, hernia nuclei pulposi or malignancy)
- Mentally incompetent (inability of a person to make or carry out important decisions regarding his or her affairs)
- No understanding of the Dutch language (written)

Patients who agree to participate sign a consent form, thereafter they will be called by a research assistant to confirm the inclusion criteria. The research assistant will register the answers to the CaFaSpA referral rule of the participant. After this telephone contact the patient will receive online questionnaires per email concerning their back pain. If email contact it is not possible, the patient will receive the questionnaires by post.

Those who do not wish to participate will be registered by gender, date of birth and the reason for not participating, such as no current low back pain, no time, etc.

### Randomisation, allocation procedure and blinding

Primary care practices are randomly allocated to either the intervention or the control group. Randomisation is stratified for number of GPs working in the primary care practice (one or two vs more than two) to ensure similar number of patients in both groups. The block randomisation schedule is computer generated and administrated by an independent person, who is not involved in patient care. It is impossible to blind patients or GPs for allocation. If a patient receives the advice of a referral to the rheumatologist, both the patient and the GP are actively involved in this referral. Also the outcome assessment is not blinded, as patients assess the outcomes themselves by filling in questionnaires. Blinded analyses of the data will take place when possible.

### Intervention

The intervention is the application of the CaFaSpA referral rule by GPs in young primary care patients with CLBP [4]. [5] (Table 1) The CaFaSpA rule is explained to GPs, first by phone and thereafter by sending written materials explaining the referral rule. To ensure that the intervention is equally applied in all participants, not the GP but a research assistant registers the answers to the referral rule from a patient. This takes place in the same telephone contact in which the inclusion and exclusion criteria are checked. The outcome of the intervention i.e. the outcome of the CaFaSpA referral rule is communicated by a letter to both the GP and the participant. If the referral rule is positive, a referral to the rheumatologist will be advised. Thereafter it is the responsibility of the GP and the patient to arrange a referral to the rheumatologist.

**Table 1:** The CaFaspA referral strategy

Applicable in patients ≥3 months back pain and age at oneset <45 years
*Inflammatory back pain*
Inflammatory back pain is considered present if at least four questions are answered with yes
-Age at onset<40 years
-Insidious onset
-Improvement with exercise
-No improvement with rest
-Pain at night (with improvement upon getting up)
*Positive family history*
A positive family history is considered present if there is a first or second degree family member with axial spondyloarthritis, Crohn’s disease, psoriasis or uveitis anterior
*Good reaction to NSAIDs*
*A* good reaction to NSAIDs is present when a patient reports a relieve in pain perception within 48 hours after receiving a NSAID
*CLBP ≥5 years*
A long low back duration is present if the duration of the back pain is 5 years or longer
If at least two out of the four referral parameters are present → a referral to the rheumatologist is advised

### Control group

Participating patients of the control group are also called by our research assistant to check the inclusion criteria and to register the answers to the CaFaSpA referral rule. No active advice regarding a referral takes place. If control group patients choose to go to their GP, they will be treated according to the Dutch College of General Practice guidelines for the management of low back pain [11].

Before the IMPACT study started we discussed with GPs the weaknesses and difficulties of our study design. We received feedback concerning the benefit of participating for GPs or patients who were randomized to the control group. It was feared that the lost to follow up would be great in the usual care group. To increase the feasibility of our study we decided to communicate the outcome of the referral rule to the control group after 4 months and thereby eliminating the contrast between the intervention and usual care group.

**Fig. 1:**
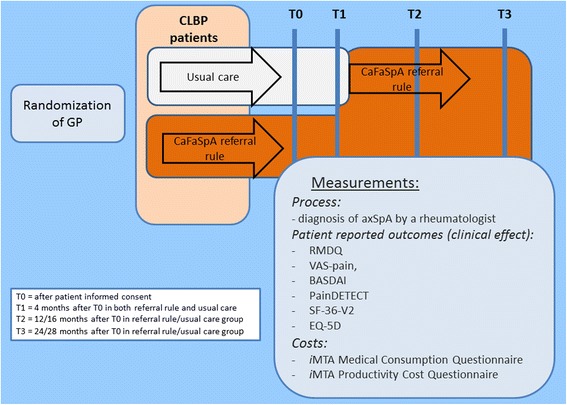
Flowchart of the IMPACT study

Therefore, four months after inclusion (after our primary outcome time point) the usual care CLBP patients and their GPs will receive a letter containing the outcome of the referral rule and an advice to refer or not refer the patient. After receiving the outcome of the referral rule the patients of the control group will be followed for two years, data will be collected after 1 and 2 years of exposure to the referral rule, this will be sixteen and twenty-eight months after inclusion of the study (Fig. 1).

### Data collection

Data collection of patient outcomes are at baseline (T0) (directly after inclusion of a patient in the study), after 4 months (T1), after 12 or 16 months (T3) and after 24 or 28 months (T4) (Fig. 1). At each time point patients will automatically receive an email with a link to online questionnaires.

### Outcome measures

The primary clinical outcome is the score on the Roland Morris Disability Questionnaire (RMDQ) after 4 months [12]. The RMDQ is a patient reported outcome and has a scale of 0 to 24. A higher score indicates a more severe disability score. The advantage of using the RMDQ as primary outcomes measure is that it is applicable in all participants as all participants are suffering from low back pain complaints. While for instance the BASDAI is only validated to use in axSpA patients. In general, the outcomes measures can be divided into three categories:

- Patient reported outcomes
  - o Disability measured by the Roland Morris Disability questionnaire
  - o Pain measured by the Visual Analogue Scale (VAS) pain [13]
  - o Health related quality of life measured by the SF-36 version 2 and EQ-5D [14, 15].
  - o Disease activity for axSpA, measured by the BASDAI [16].
  - o Neuropathic components related to back pain measured by the painDETECT [17]
- Process
  - o Diagnosis of axial spondyloarthritis made by rheumatologists, which is verified by hospital records.
- Costs
  - o Loss of work-productivity measured by work participation (*i*MTA Productivity Cost Questionnaire iPCQ)) [18]
  - o Health care resources use measured by the *i*MTA Medical Consumption Questionnaire [19]

### Compliance

The compliance of patients is optimized by sending up to three reminders emails, asking the patient to fill out the online questionnaires. If the patient still has not completed the questionnaires the research assistant will contact the patient by telephone and the questionnaires are sent to the patient by post.

### Sample size

For the power calculation we assumed a difference of 2.5 points on the RMDQ at four months between the referral rule and usual care group. These 2.5 points are the clinically significant difference, found in previous studies [20, 21]. The SD of the RMDQ is 6.0 based on data observed in the previous CaFaSpA 2 study [4]. Detection of this 2.5 point improvement in a randomized trial would require 180 patients per group, using a two-sided α of 0.05 and power of 0.80.

Patient in the intervention group who have a negative result of the referral strategy (i.e. no referral to the rheumatologist) will receive the same treatment as the usual care group. Therefore, the effect of the referral strategy can only be assessed in patients with a positive result of the referral strategy. From the previous CaFaSpA studies we know that around 50 % of the participating patients will have a positive result. Therefore 360 patients(180 *x*2)would be required for 80 % power.

Further, the sample size was adjusted for cluster randomization based on an intra-cluster correlation coefficient of 0.05 and an average cluster size of 16 [22]. [23] The average cluster size is based on data of the CaFaSpA 1 and 2 studies, with on average 16 participating patients per GP. With these findings we can calculate the design effect; design effect = 1 + (16–1) * 0.05 = 1.75

**Fig. 2:**
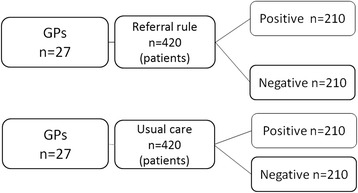
Sample size calculation taking clustering into account (ICC of 0.05)

Multiplying 360 patients by the design effect of 1.75 implies that a total of 630 patients must be included in this study. If a lost to follow up of 25 % is taken into account 840 patients need to be enrolled. Assuming 16 CLBP patients per GP, implies that 54 GPs (840/16) need to be randomized (Fig. 2). We expect that 16 patient per GP practice will participate, if the number of participating patients per practice is smaller, for example only 6, this will lead to a smaller design effect (1.25) and a total of only 600 patients should be enrolled to create sufficient power.

### Data analysis

Effects at 4 months and the process evaluation will be analysed according to the intention-to-treat principle. The baseline characteristics of the patients will be summarized by randomisation group, reported as mean (standard deviation) or median (interquartile range) for continuous variables and count (percent) for categorical variables.

As this is a cluster-randomized trial mixed effect regression analysis will be used to compare the mean RMDQ score after four months between the intervention and usual care group. Fixed effects include allocation group and result of the referral strategy (referral y/n). As the effect of the referral strategy is expected in the subgroup of patient with a referral advice, an interaction term between allocation group and result of the referral strategy will also be included. A random intercept will be included adjusting for clustering [24, 25]. This random intercept stand for the effect of different primary care practices (i.e. clusters).

For the secondary outcomes we will again use a mixed effect regression analysis to estimate the effect of the use of the referral strategy after 4 months on process level (i.e. the number of axSpA diagnoses by a rheumatologists), pain (VAS pain), quality of life (SF-36 version 2 and EQ-5D) and disease activity (BASDAI). We will use linear regression for continuous outcomes and logistic regression for dichotomous outcomes. Similar to the primary outcome analysis an interaction term and random intercept will be used to take into account interaction between allocation group and referral strategy and clustering. We intended to assess the effect of using the referral strategy also at 12 and 24 months. However, patients in the control group receive also an advice based on the referral rule after 4 months. Contract between the intervention and control group is no longer present. Therefore, individual trajectories will be modeled using random effect regression with patient outcome as the dependent variable and time as covariate and a random intercept for patient.

For the cost evaluation we will compare costs before and after the application of the referral strategy. We will consider costs of provided health care and costs due to loss of work-productivity. In order to calculate costs, the volume of care will be linked to the actual, integrated cost prices per medical service [26].

A *p*-value of <0.05 will be considered statistically significant. Statistical analyses will be undertaken with STATA.

## Discussion

This evaluation aims to assess the effects on patient outcomes, processes and costs of a referral strategy for axSpA in young primary care patients with CLBP by comparing the use of the strategy with usual care. The study started in July 2014 and the first results are expected in May 2016.

There are only a very few impact studies in the field of prognostic research. In a recent review there were 61 development studies and only 2 (3 %) of them also had an impact evaluation [27], an essential step to asses clinical effectiveness and costs.

The main strength of this study is that it provides information on the process outcome (referral to the rheumatologists and result of the diagnostic process) and on the patient outcomes (pain and quality of life). The combination of process and patients outcomes allows for a better interpretation of findings. An effect in process does not necessarily results in improved patient outcomes. Possible absence of effect in patient outcomes, on the other hand, may be the result of insufficient improvement in the process. Further, the study measures the impact of a validated referral strategy. The referral strategy has already shown to discriminating axSpA patients from other CLBP patients [4]. We have chosen to only test the impact of the CaFaSpA referral strategy as this is the cheapest and the most feasible strategy for primary care of all proposed referral strategies for axSpA.

A weakness of the study is that the contrast between intervention and control groups disappears after 4 months, as patients of the control group are also provided with the advice of the referral strategy. Nevertheless our primary outcome is assessed before the contract between intervention and control group is eliminated. We expect that 4 months is a sufficient period to achieve a substantial improvement in the primary outcome by using the referral strategy in the intervention group. For other outcomes we can use a before after design within patients which is acceptable as the back pain complaints are chronic. Another potential weakness is that patients are selected by a registry, rather than only actively care seeking patients are invited to participate. This can lead to a lower participation rate of the invited patients and a potential selection bias of only severe cases of low back pain. However in the prior CaFaSpA studies, the same approach to select participants by the GP register was conducted and this didn’t result in a more severe low back pain study population [4, 5]. In both CaFaSpA studies was the VAS pain comparable with other low back pain cohorts. One last limitation of this study is that the intervention i.e. the CaFaSpA referral rule is applied by research assistant not by GPs themselves, this pragmatic approach can influence the outcomes of the study. We have chosen for this pragmatic approach as GPs pointed before the start of study that it is often too busy during consultation hour to properly ask the questions of the referral rule and that the application of the referral rule would not be uniform but heavenly time dependent.

If this study succeeds in demonstrating an impact of applying the referral strategy for axSpA in young CLBP patients, the potential benefit may be substantial. The care provided to CLBP patients can be improved, it will be easier for GPs to refer the CLBP patient with a high risk for axSpA to the rheumatologist. And an earlier diagnosis of axSpA has favorable outcomes, as several studies have shown that an effective treatment in axSpA patients results in a lower disease activity, improved quality of life and enhanced work participation [28, 29]. And finally the gain for society; CLBP is a great socio-economic burden for society. When one of the causes for CLBP is recognized earlier and subsequently diagnosed and treated in an earlier stage this can lead to decreased sick leave due to back pain and increased work productivity.

## Abbreviations

AxSpA, Axial Spondyloarthritis; BASDAI, Bath Ankylosing Spondylitis Disease Activity Index; CaFaSpA, Case Finding Axial Spondyloarthritis; CLBP, Chronic Low Back Pain; EQ-5D, EuroQol five dimensions; GP, general practioner; ICPC, International Classification Primary Care; iMCQ, IMTA Medical Consumption Questionnaire; iPCQ, IMTA Productivity Costs Questionnaire; LBP, low back pain; RMDQ, Roland Morris Disability Questionnaire; SF-36, Short Form Health Survey; VAS, Visual Analogue Scale; YLD, Years Lived with Disability
